# A Novel 3D Multilateration Sensor Using Distributed Ultrasonic Beacons for Indoor Navigation

**DOI:** 10.3390/s16101637

**Published:** 2016-10-08

**Authors:** Rohan Kapoor, Subramanian Ramasamy, Alessandro Gardi, Chad Bieber, Larry Silverberg, Roberto Sabatini

**Affiliations:** 1 School of Engineering, RMIT University, Aerospace and Aviation Discipline Melbourne, Melbourne VIC 3000, Australia; s3572380@student.rmit.adu.au (R.K.); subramanian.ramasamy@rmit.edu.au (S.R.); roberto.sabatini@rmit.edu.au (R.S.); 2 Mechanical and Aerospace Engineering, NC State University, Raleigh, NC 27695, USA; cbieber@ida.org (C.B.); lmsilver@ncsu.edu (L.S.)

**Keywords:** navigation, overdetermined system, trilateration, ultrasonics, distributed sensing

## Abstract

Navigation and guidance systems are a critical part of any autonomous vehicle. In this paper, a novel sensor grid using 40 KHz ultrasonic transmitters is presented for adoption in indoor 3D positioning applications. In the proposed technique, a vehicle measures the arrival time of incoming ultrasonic signals and calculates the position without broadcasting to the grid. This system allows for conducting silent or covert operations and can also be used for the simultaneous navigation of a large number of vehicles. The transmitters and receivers employed are first described. Transmission lobe patterns and receiver directionality determine the geometry of transmitter clusters. Range and accuracy of measurements dictate the number of sensors required to navigate in a given volume. Laboratory experiments were performed in which a small array of transmitters was set up and the sensor system was tested for position accuracy. The prototype system is shown to have a 1-sigma position error of about 16 cm, with errors between 7 and 11 cm in the local horizontal coordinates. This research work provides foundations for the future development of ultrasonic navigation sensors for a variety of autonomous vehicle applications.

## 1. Introduction

In recent years, there has been an increasing research focus on developing and improving navigation systems for air, ground, and underwater vehicles. Navigation systems including the Global Navigation Satellite System (GNSS) are not possible to use underwater, and in several air and ground vehicle applications, it is prone to data degradations or the complete loss of signal due to multipath effects, interference, and antenna obscuration [1,2,3,4,5]. Hence, GNSS signals are not reliable in urban landscapes comprised of tall buildings and their performance further deteriorates in an indoor environment. Additionally, GNSS systems have an accuracy of a few meters, which is not suitable for most indoor navigation applications.

Multilateration is a method used to determine the position of an object based on simultaneous range measurements from three or more anchors located at known positions [6]. If the number of anchors used is three, it becomes a case of trilateration. Trilateration has been implemented in ultrasonics-based localization systems like Active Bat [7], Cricket [8], Dolphin [9], and Millibots [10]. Alternate localization methods like Received Signal Strength (RSS), triangulation, and Time Difference of Arrival (TDOA) have been thoroughly investigated in the recent past. RSS has insufficient precision [11] and lower resolution [12] for an indoor environment. Triangulation requires expensive hardware like directional antennas and the equations employed are more complex than trilateration equations [13]. TDOA computations require the sharing of data between receivers, which in turn dictates bandwidth and power requirements [14]. Furthermore, TDOA calculations involve the intersection of hyperbolic surfaces, which increases the computational complexity. Ultrasonic sensors are relatively inexpensive and robust against environmental noise. This makes them preferable to other location techniques that employ visual, tactile, and magnetic systems [15].

Based on these premises, this research focuses on the development and testing of an in-door 3D ultrasonic positioning system using multilateration for real-time dynamic platform applications. The proposed system is tested for positioning accuracy in relevant static and dynamic case studies, including both numerical simulations and experimental tests. After discussing the principles of multilateration, a suitable iterative algorithm is introduced for over-determined multilateration problems. The results of the numerical simulations and experiments corroborate the validity of the proposed sensor architecture. After discussing the key research findings, the paper sums up with conclusions and recommendations for future work.

## 2. Multilateration Principles

**Figure 1:**
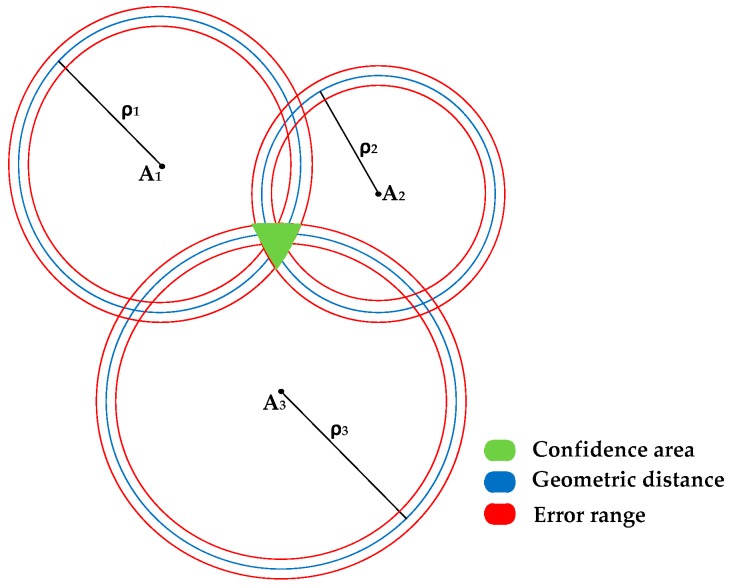
Multilateration position calculation.

In 2D space, trilateration requires at least three measured distances between anchors and a node, the location of which is to be determined. The anchors should not be collinear and their position should be fixed and known for higher accuracy. After the measurements are acquired, the location of the node can be determined as the intersection of three circumferences whose geometric centres coincide with the anchor positions. The measured distance is represented by the radii from the anchors. Figure 1 depicts the trilateration principle in 2D involving three anchors and associated spheres of different radii, depicting the range from each anchor. The confidence area, depicted in green, is the intersection of all three circles. In 3D space, a minimum of four measurements are required for multilateration. The node is located at the intersection of spheres, with anchors fixed at their geometric centres.

The fundamental principle adopted in multilateration systems is to measure the ranges between the receiver (node) and simultaneously observed transmitters (anchors). The equation for geometric distance between the receiver and the transmitter can be written as:

(1) $$\rho \left(t\right)=\;\sqrt{{\left(x^{a}-\;x_{k}\right)}^{2}+\;{\left(y^{a}-\;y_{k}\right)}^{2}+\;{\left(z^{a}-\;z_{k}\right)}^{2}}$$

where ($x_{k}$, $y_{k},z_{k})$ are the co-ordinates of the receiver and ($x^{a}$, $y^{a}$, $z^{a}$) are the transmitter coordinates. A purely analytical solution for position in 3D space can be found with a system of three equations with three unknowns:

(2) $$\rho_{n}\left(t\right)=\sqrt{{\left(x^{a}-\;x_{k}\right)}^{2}+\;{\left(y^{a}-\;y_{k}\right)}^{2}+\;{\left(z^{a}-\;z_{k}\right)}^{2}}\;\left(n\;=\;1,\;2,\;3\right)$$

This would provide a uniquely constrained system with two solutions. However, the navigation solution calculated with this system of equations does not account for measurement errors, for a non-functional anchor, or for an anchor not detected due to being either out of the detection range or because of an obstruction. Errors in the measurements can considerably inflate one or more of the confidence bands shown in Figure 1 and this would introduce errors in the position estimates. Additionally, there are certain constraints on the arrangement of anchors (e.g., a combination of three collinear anchors), which can lead to singularities in the position computation [16]. Finally, if any of the signals are degraded or lost, the system becomes underdetermined and hence not solvable analytically.

In the proposed ultrasonic multilateration system, each range measurement is affected by a number of errors, the most significant being the time errors of the receiver and transmitter clocks. Since the range measurements are affected by these errors, they differ from the actual geometric distance corresponding to the epochs of signal transmission and reception. The general range equation is given by:

(3) $${\rho_{k}}^{t}\left(t_{k}\right)=\left(t_{k}-t^{t}\right)v$$

where ${\rho_{k}}^{t}$ is the actual measurement, $t_{k}$ is the nominal time of the receiver clock k at reception, $t^{t}$ is the nominal time of the transmitter clock s at emission, and v is the speed of sound. The clock biases are modeled using the following expressions:

(4) $$t_{r,\;k}=\;t_{k}+dt_{k}$$

(5) $$\;{t_{k}}^{t}=\;t^{t}+\;dt^{t}$$

where r denotes the true time and the terms $dt_{k}$ and $dt^{t}$ represent the receiver and transmitter clock errors respectively. Taking these errors and biases into account, the complete expression for the single range measurement becomes:

(6) $${R_{k}}^{t}\left(t_{k}\right)={\rho_{k}}^{t}\left(t_{r,k}\right)-\;\left(dt_{k}\;-dt^{t}\right)v+\;{P_{k,t}}^{t}\left(t_{k}\right)+{d_{k,T}}^{t}\left(t_{k}\right)+\;{d_{t}}^{t}\left(t_{k}\right)+d_{k,t}\left(t_{k}\right)\;+\;\varepsilon_{t}$$

where:

${\rho_{k}}^{t}\left(t_{r,k}\right)$ = geometric distance;
${P_{k,t}}^{t}\left(t_{k}\right)$ = propagation delay in air (standard conditions);
$d_{k,t}\left(t_{k}\right)$ = receiver clock error;
${d_{t}}^{t}\left(t_{k}\right)$ = transmitter clock error;
${d_{k,T}}^{t}\left(t_{k}\right)$ = multipath error;
$\varepsilon_{t}$ = random measurement noise.

The multipath error depends on the geometry of the transmitter and the receiver with respect to the surrounding reflective surfaces.

The coordinates of the receiver as well as the timing information are derived from the simultaneous observation of four (or more) transmitters. Assuming a constant clock error $dt_{k}$ for measurements to any transmitter and neglecting all other error terms, the following system of equations are obtained:

(7) $${R_{k}}^{n}\left(t\right)=\sqrt{{\left(x^{n}-\;x_{k}\right)}^{2}+\;{\left(y^{n}-\;y_{k}\right)}^{2}+\;{\left(z^{n}-\;z_{k}\right)}^{2}}+vdt_{k}\;\left(n\;=\;1,\;2,\;3,\;4\right)\;$$

Considering this system of equations, a minimum of four transmitters is required to yield four equations, which are solved to provide the positioning solution.

### Multilateration Algorithm

Various algorithms were considered and evaluated for implementation in our ultrasonic sensor system [17,18,19,20,21]. These included both analytical trilateration and recursive least squares multilateration techniques. Both approaches gave similar results in most of the cases. However, for over determined system configurations, the multilateration algorithms were found to give more accurate and robust positioning solutions. Therefore, to make our system more robust against data losses and measurement errors, our research focused on efficient algorithms that could accommodate redundant measurements both in a static and dynamic environment. In [17], a linear algebraic method is presented to compute an optimal solution for over determined multilateration problems. However, this algorithm is not suitable for dynamic platform applications. Considering the nature of the ultrasonic sensor system proposed in our research, a recursive least squares algorithm [18,19] was selected for the ultrasonic positioning system.

When more than four transmitters are being observed, the problem is over-determined and can be solved in a least squares sense to yield an optimal estimate of the receiver location. In practice, the solution is obtained iteratively starting from an initial guess of the receiver position $\left(x_{0},\;y_{0},\;z_{0}\right)$. In this case, we have:

(8) $$x=x_{0}+\Delta x$$

(9) $$y=y_{0}+\Delta y$$

(10) $$z=z_{0}+\Delta z$$

where Δx, Δy, and Δz are the differences between the true solution and the initial guesses (corrections to the nominal values). To correct the initial guess, a linear (first-order Taylor) expansion of $\rho_{k}$ is adopted. Let n denote a preliminary best estimate value. The linearised equation is:

(11) $$-\frac{x_{n}-x^{s}}{{P_{n}}^{s}}\Delta x_{k}-\frac{y_{n}-y^{s}}{{P_{n}}^{s}}\Delta y_{k}-\frac{z_{n}-z^{s}}{{P_{n}}^{s}}\Delta z_{k}+\Delta dt_{k}=\Delta R^{s}$$

where ${R_{n}}^{s}$ is the nominal range measurement to the *s*th transmitter and $\Delta R^{s}$ is the difference between the actual and nominal range measurements. In addition to the ranging errors that affect the sensor position accuracy, the relative geometry of the transmitters and the receiver affects the estimation. The four linearised equations considering four transmitters are given by:

(12) $$\left[\begin{array}{c} \begin{array}{c} \Delta R^{1}\\ \Delta R^{2} \end{array}\\ \Delta R^{3}\\ \Delta R^{4} \end{array}\right]=\left[\begin{array}{cccc} d_{11} & d_{12} & d_{13} & 1\\ d_{21} & d_{22} & d_{23} & 1\\ d_{31} & d_{32} & d_{33} & 1\\ d_{41} & d_{42} & d_{44} & 1 \end{array}\right]\;\left[\begin{array}{c} \Delta x_{k}\\ \Delta y_{k}\\ \begin{array}{c} \Delta z_{k}\\ \Delta dt_{k} \end{array} \end{array}\right]+\;\left[\begin{array}{c} \varepsilon_{1}\\ \varepsilon_{2}\\ \begin{array}{c} \varepsilon_{3}\\ \varepsilon_{4} \end{array} \end{array}\right]$$

where $d_{ij}$ is the direction cosine of the angle between the Line of Sight (LoS) to the *i*th transmitter and the *j*th co-ordinate and $\varepsilon_{i}$ represents the measurement and other errors. Hence the solution is given by:

(13) $$\left[\begin{array}{c} \Delta x_{k}\\ \Delta y_{k}\\ \begin{array}{c} \Delta z_{k}\\ \Delta dt_{k} \end{array} \end{array}\right]=\;-\;{\left(B^{T}{\sigma_{0}}^{2}\;R_{\rho }\;B\right)}^{-1}\;B^{T}\;{\sigma_{0}}^{2}\;R_{\rho }\;\left[\begin{array}{cccc} \Delta P^{1} & \Delta P^{2} & \Delta P^{3} & \Delta P^{4} \end{array}\right]$$

where B is the matrix of coefficients of the linear set of equations, $R_{\rho }$ is the covariance matrix of the pseudorange errors, and ${\sigma_{0}}^{2}$ is a scale factor known as the a priori variance of unit weight.

## 3. Numerical Simulation

**Figure 2:**
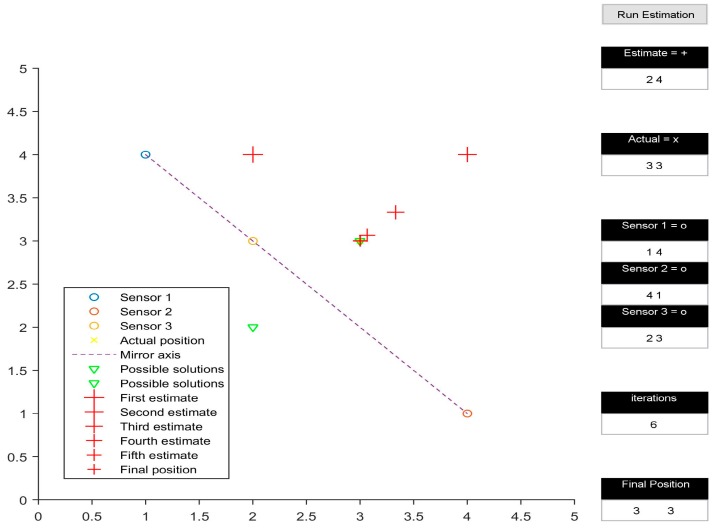
Collinear convergence.

**Figure 3:**
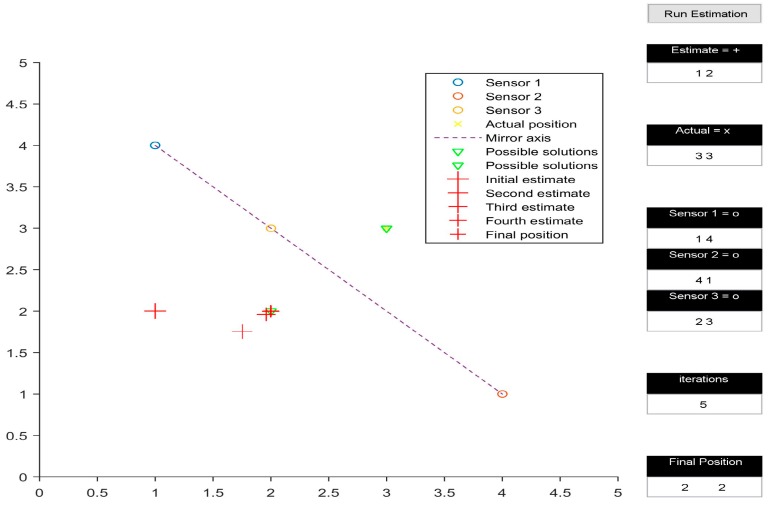
Mirroring.

**Figure 4:**
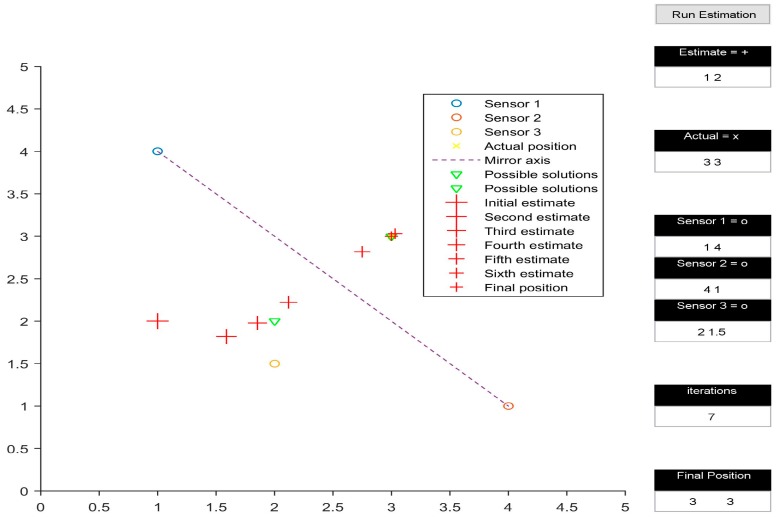
Non-collinear convergence.

**Figure 5:**
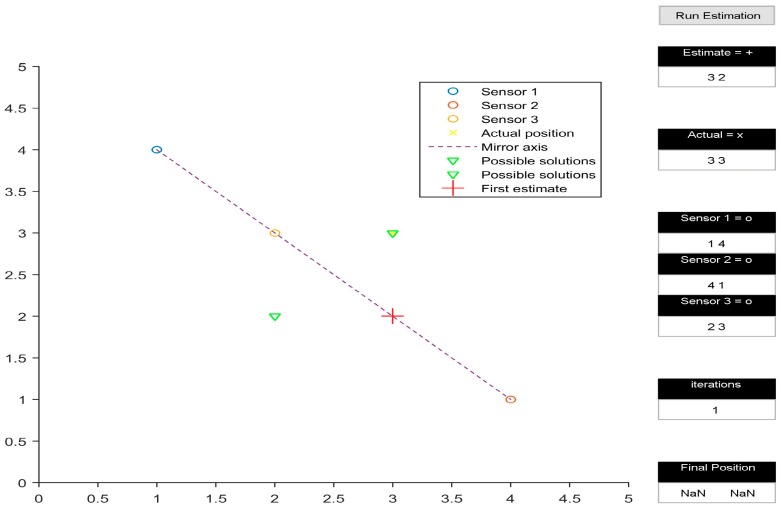
Singularity.

The multilateration algorithm was tested in a representative simulation environment. This activity allowed us to define an effective layout of the beacons and to estimate the navigation performance achievable by the system in the intended application. In particular, a user interface was developed in MATLAB to test the system in 2D space. The test area was assumed to be a 5 by 5 unit rectangular space. The position of the three transmitters within the test area could be easily adjusted by the user. Additionally, the initial guess of the position of the receiver could also be defined by the user. Figure 2, Figure 3, Figure 4 and Figure 5 depict various simulation test cases. The number of iterations required to obtain the final estimate, as well as their depiction in the test area, are displayed in the interface.

The simulations were performed on four test cases. The three transmitters are placed at (1, 4), (4, 1) and (2, 3) for all cases except Figure 4. Figure 2 presents the test case in which the presented algorithm calculates the coordinates of the receiver correctly, taking six iterations to do so. It is interesting to note how the initial estimate of the receiver position determines the final output of the algorithm, subject to the geometric arrangement of transmitters. In Figure 2 and Figure 3, all three transmitters being collinear, the final estimate is dependent upon the initially estimated position in 2D space. When the 2D space is divided into two planes by the line joining the transmitters, the final estimate is calculated correctly only if the initial estimate is in the same plane as the actual receiver position, as shown in Figure 2. Otherwise, the calculated position is the mirror image of the receiver position, with the line joining the transmitters acting as the mirror axis.

Figure 3 shows the calculated receiver position to be (2, 2), which is the mirror image of (3, 3) with respect to the line joining (1, 4), (4, 1), and (2, 3). However, the mirroring disappears when the transmitters are rearranged in a non-collinear configuration, as shown in Figure 4. Another observation made while analysing the simulation results occurred when the initial estimate of the receiver’s position was in line with the already collinear transmitter configuration (Figure 5); this led to a mathematical singularity and hence the algorithm failed to proceed.

An in-depth analysis of all simulation results supported the design and development of the experimental setup for validation of the system. The experimental positioning system was set up and various experiments were conducted to test the 3D positioning system for robustness to noise, sensors not being detected due to an obstruction, or sensors being out of range. The real-time positioning capability of a moving receiver was also tested.

## 4. Experimental Verification

**Figure 6:**
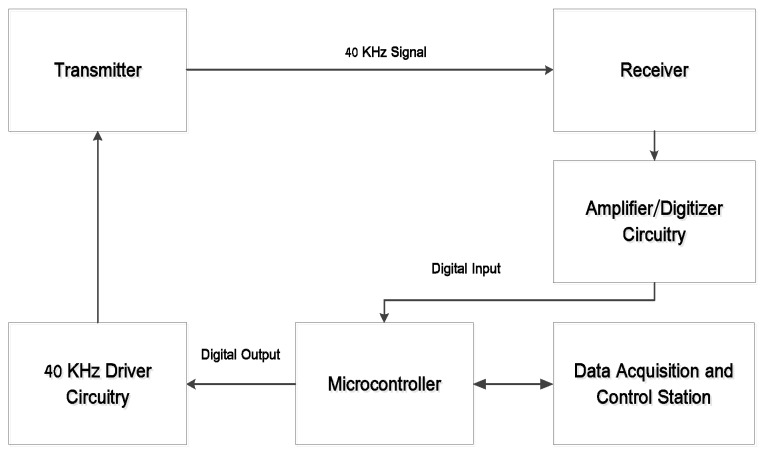
3D positioning system schematic.

The conceptual schematic of a 3D positioning system based on ultrasonic beacons is shown in Figure 6. The transmitters are connected to an amplifier and timing circuit, which is configured to dispatch 200 microsecond (eight cycles) bursts. The transmitter circuit receives a trigger from the microcontroller, which initiates the timing of the ultrasonic signals being sent. The receiver circuit consists of an amplifier and sends a binary 1 value to the microcontroller upon receiving the ultrasonic signal. Errors due to multipath have been neglected in the positioning system.

**Figure 7:**
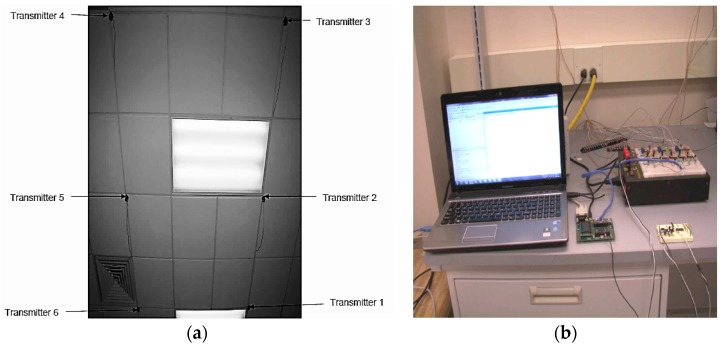
Layout of the experimental setup, consisting of: (**a**) transmitters mounted on the ceiling; (**b**) 3D positioning system.

An experimental setup was built in an indoor environment for testing of the 3D positioning system. The setup consisted of six 40 KHz transmitters mounted in a 2.4 m by 0.9 m grid on the ceiling (Figure 7a) and a receiver circuit connected through a microcontroller to the computer (Figure 7b). Initially, calibration of the Time-of-Flight measurements was conducted on a test bench. The conversion factor for converting the Time-of-Flight measurements to distance was obtained from the calibration process. This conversion factor was subsequently introduced in the positioning algorithms for post processing as well as for real time dynamic positioning algorithms. The microcontroller employed was QSK62P Plus [22]. QSK62P Plus is a 16-Bit microcontroller that has Serial Input/Output (I/O) that supports Serial Peripheral Interface (SPI) bus to the computer. The QSK62P microcontroller was chosen specifically among the available options as it had the required number of I/O and SPI ports. The frequency of the microcontroller was 3 MHz for time-of-flight measurements. This allowed for very high-resolution distance measurements. However, there are certain offsets introduced, both constant and variable, due to the time delays in various stages of the 3D positioning circuitry, discussed further in Section 4.1.

The timing data, which was in the form of unprocessed microcontroller ticks, was sent to the computer through the serial port interface. The data was received by MATLAB for further processing to calculate the receiver coordinates.

### 4.1. Sources of Error

**Figure 8:**
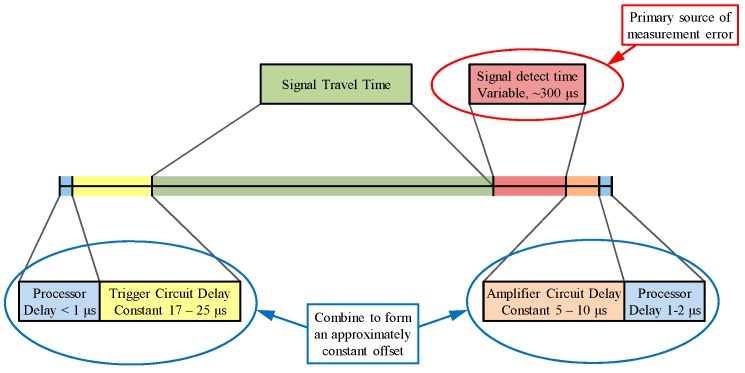
Signal timeline and sources of error (not to scale).

There are various sources of error in the positioning system resulting primarily from hardware limitations. As shown in Figure 8, a constant offset of 40 microseconds was introduced due to delays in the microcontrollers and the analogue circuitry. Additionally, the receiver circuit could cause a delay of up to 300 microseconds in detecting the received signal, leading to an error of about 9 cm. Treating the total clock error as an unknown in the iterative least square algorithms described in Section 2, this error can be significantly reduced (subject to the assumption of a constant clock error for all simultaneous observations).

### 4.2. Results

The 3D positioning system was tested in terms of its linear and angular range performances, effects of an obstruction in the path of the transmitter-receiver pair, and real time positioning of a moving receiver. The experimental setup consisted of an area of 3.6 by 2.4 m and a height of about 2.8 m.

**Figure 9:**
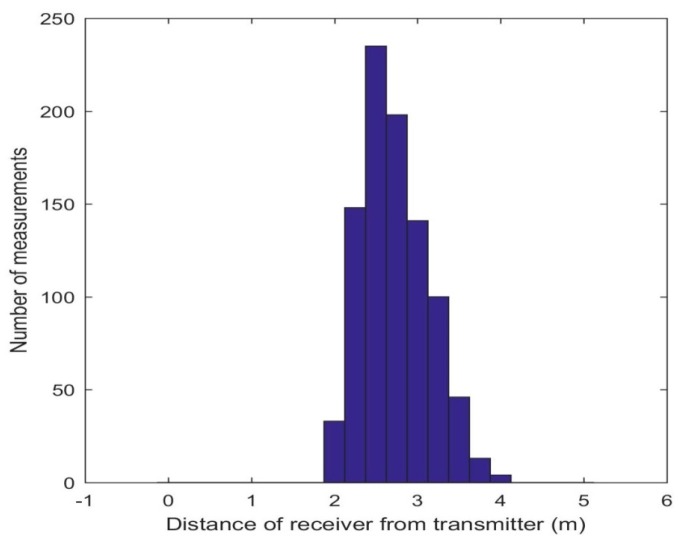
Linear range distribution of the ultrasonic sensors.

Based on component characteristics, signal power, and on nominal background noise figures, the calculated nominal range of the ultrasonic system was about 3 m, though measurements up to 4 m were recorded. The range could be extended by improving the circuitry or procuring off the shelf commercially available hardware. With a higher range, a larger area could be covered with fewer anchors. The limited range helped to mitigate the effects of errors introduced due to multi-path reflections. The origin of the global coordinate system corresponded to a point on the floor with the *Z* axis perpendicular and the *X*/*Y* axes parallel to the transmitter arrangement. A total of 256 points were measured and each point was an average of three readings. Eliminating the points where the coordinates could not be calculated due to range limitations, the number of points reduced to 196. The linear range distribution obtained is shown in Figure 9.

**Figure 10:**
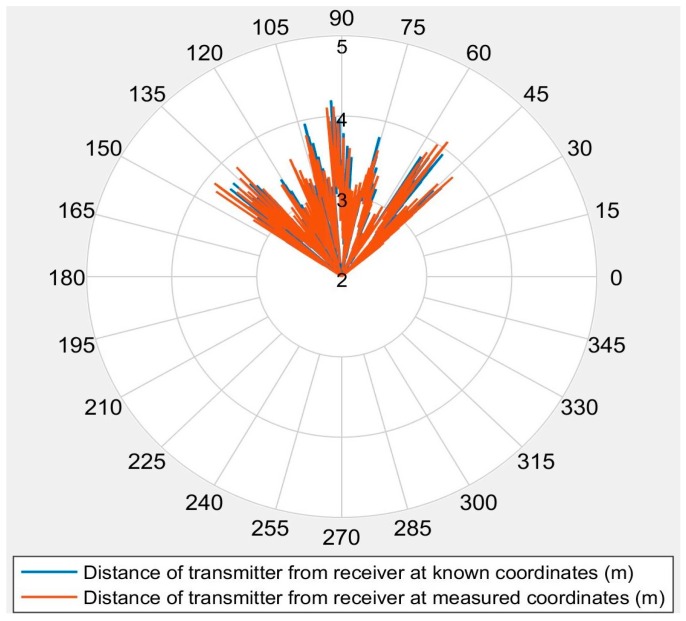
Angular range of the ultrasonic sensors.

The angular range of the ultrasonic sensors is an important parameter in determining the configuration of the transmitters for a given volume. Ultrasonic sensor datasheets normally state that the sensors have a conical range map, with an opening angle of about 45° [23]. Due to its high relevance for operational implementations, the angular range of all six transmitters was thoroughly monitored during the entire experimental campaign of the 3D positioning system. Since all transmitters were of the same configuration, they were expected to behave similarly. Coordinate transformations from the global reference system to coordinate systems with the origin at the receiver were performed. Although there was no separate experimental investigation performed to assess the angular detection range of the ultrasonic sensors, the angular range data for all receiver positions from the experiments showed a coverage exceeding 45°, as shown in Figure 10, which is in accordance with the sensor datasheet.

To analyse the effect of obstruction on the transmitter-receiver pair, a balloon with about a 22 cm diameter was used. The balloon was made to traverse a fixed path parallel to the *X*-axis of the global coordinate system at three different heights (*Z* coordinates), i.e., 0.9, 1.4, and 2.2 m, respectively. The transmitter ticks (raw microcontroller data for distance) for each of the transmitter-receiver pairs were measured at regular intervals. The ticks do show an effect as the balloon passes over the transmitter-receiver pairs. The impact of obstruction varies for the transmitters, owing to the dissimilar transmitter-receiver orientations and the distance of the obstruction from the transmitters. However, the transmitter ticks change for every transmitter when the obstruction comes above or near the transmitter-receiver line of sight, when visualized in the *X*-*Z* plane. The sensors, all being in range, should never give a zero tick. Hence, the ticks that were zero can solely be attributed to the obstruction. It is interesting to note that the ticks tended to increase in value before going to zero in most of the cases. This suggests some sort of bending of sound waves off the surface of the obstruction before they reach the receiver. There needs to be more extensive experiments before conclusive evidence can be provided about the impact of obstructions. However, obstructions can have an impact on system performance and their presence should be avoided for more efficient performance of the 3D positioning system.

**Figure 11:**
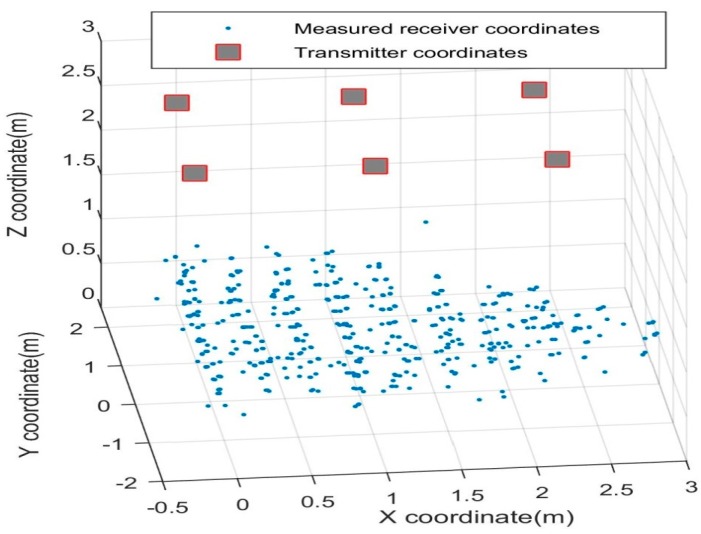
3D distribution of the measured receiver coordinates.

**Figure 12:**
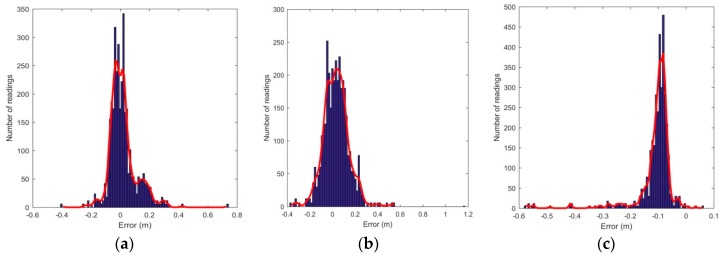
Errors in the measured receiver coordinates. (**a**) Error in *X* coordinates; (**b**) Error in *Y* coordinates; (**c**) Error in *Z* coordinates.

Figure 11 shows the sample readings taken for static analysis of the 3D positioning system, with the transmitter arrangement depicted as well. The errors in calculated position with respect to the actual coordinates are also analysed. The error distribution is observed to be roughly Gaussian, as shown in Figure 12. Table 1 lists the statistical data (mean and standard deviation) of the errors in all three axes. It is observed that the error in the *Z* coordinate is considerably higher than in the *X* or *Y* coordinates, which is intuitive as the transmitter grid is arranged in an *X*-*Y* plane. Table 2 lists the position errors.

**Figure 13:**
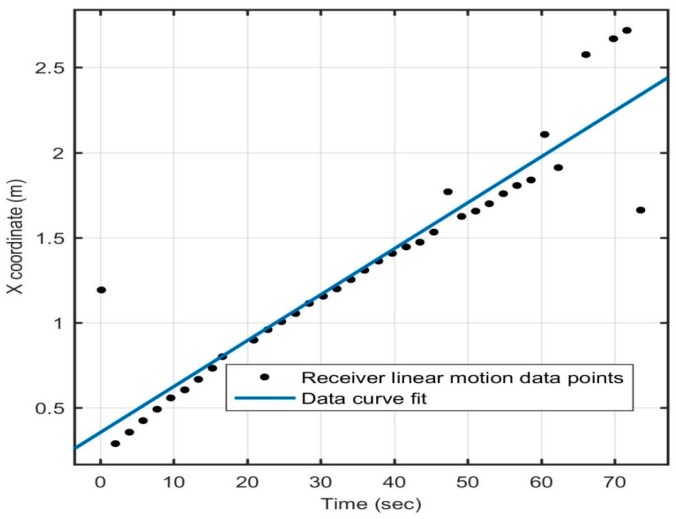
Straight line motion.

**Figure 14:**
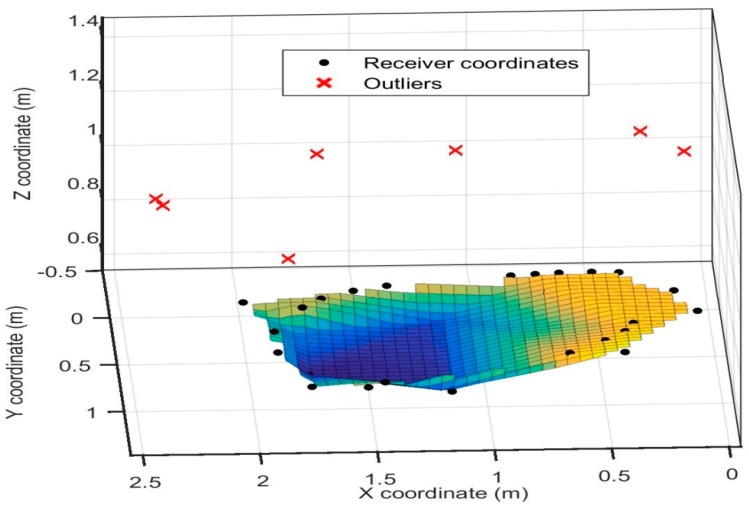
Motion in a circle.

Dynamic tests were also performed on the receiver to evaluate the positioning system’s performance as the receiver moved within the test volume. The receiver was moved at a speed of about 3 cm/s in a straight line and in a circle, as shown in Figure 13 and Figure 14, respectively, and the motion of the receiver was captured by the 3D positioning system in real time. Readings were taken for straight line motion and for motion in a circle. An outlier point was found to be erroneous in all *X*, *Y*, and *Z* coordinates, as the coordinates were coupled. There was a latency of about 1.98 s between two successive position calculations, which was primarily due to the time lag introduced because of data transmission to the computer for the position computation. This explains the presence of about 15% outliers in dynamic tests.

## 5. Conclusions and Future Work

This paper demonstrated the feasibility of an indoor 3D positioning system based on multilateration using ultrasonic beacons. This approach is particularly suitable for navigation of autonomous vehicles in indoor and urban environments and is optimally fit to overcome the limitations of radionavigation and Global Navigation Satellite Systems (GNSS) in these scenarios [2,5]. As the considered ultrasonic sensors are relatively inexpensive and easy to set up, the proposed positioning system has the potential to be scaled up to cover a bigger volume, opening up various operational applications. The inherent nature of the slow propagating speed of ultrasonic waves allows for higher signal resolution and thus leads to increased accuracy in position estimations. The system was tested for robustness in the presence of noise, obstructions in line of sight of sensors, sensors being out of range, and failures. Having an overdetermined system for position estimation mitigates many of the undesirable effects, thereby increasing the system robustness.

Future developments of the ultrasonic positioning system will include the implementation of improved circuitry for faster response, lower false-alarm rate in dynamic conditions, and extended range. This will be possible by adopting robust and fully decentralized position estimation methods capable of reducing outliers due to noise and multipath. The sensor system will also be tested in dynamic configurations, comprising of moving anchors, and attitude determination capabilities will be developed [24,25]. These developments will support further applications of the proposed ultrasonic positioning system beyond indoor navigation to include a variety of aerial, ground, and underwater vehicles.

## Figures and Tables

**Table 1:** *X*, *Y,* and *Z* coordinates error statistics.

Coordinates	Mean (cm)	Standard Deviation (cm)
*X*	1.83	9.45
*Y*	3.35	11.28
*Z*	−10.97	7.01

**Table 2:** Position error statistics.

Mean (cm)	Standard Deviation (cm)
17.51	16.30
